# Brassinosteroid Signaling Converges With Auxin-Mediated C3H17 to Regulate Xylem Formation in *Populus*

**DOI:** 10.3389/fpls.2020.586014

**Published:** 2020-10-27

**Authors:** Xianfeng Tang, Congpeng Wang, Yu Liu, Guo He, Nana Ma, Guohua Chai, Shengjun Li, Hua Xu, Gongke Zhou

**Affiliations:** ^1^Key Laboratory of Biofuels, Shandong Provincial Key Laboratory of Energy Genetics, Shandong Institute of Energy Technology, Qingdao Institute of Bioenergy and Bioprocess Technology, Chinese Academy of Sciences, Qingdao, China; ^2^College of Resources and Environment, Qingdao Agricultural University, Qingdao, China; ^3^State Key Laboratory of Crop Biology, College of Life Science, Shandong Agricultural University, Tai’an, China

**Keywords:** populus, auxin, PdC3H17, brassinosteroids, xylem formation

## Abstract

Brassinosteroid (BR) signaling has long been reported to have an effect on xylem development, but the detailed mechanism remains unclear, especially in tree species. In this study, we find PdC3H17, which was demonstrated to mediate xylem formation driven by auxin in our previous report, is also involved in BR-promoted xylem development. Y1H analysis, EMSA, and transcription activation assay confirmed that *PdC3H17* was directly targeted by PdBES1, which is a key transcriptional regulator in BR signaling. Tissue specificity expression analysis and *in situ* assay revealed that *PdC3H17* had an overlapping expression profile with *PdBES1*. Hormone treatment examinations verified that xylem phenotypes in *PdC3H17* transgenic plants, which were readily apparent in normal condition, were attenuated by treatment with either brassinolide or the BR biosynthesis inhibitor propiconazole. The subsequent quantitative real-time polymerase chain reaction (qRT-PCR) analyses further revealed that BR converged with PdC3H17 to influence transcription of downstream xylem-related genes. Additionally, the enhancement of xylem differentiation by auxin in *PdC3H17* overexpression plants was significantly attenuated compared with wild-type and dominant negative plants due to BR deficiency, which suggested that the BR- and auxin-responsive gene *PdC3H17* acted as an mediation of these two hormones to facilitate xylem development. Taken together, our results demonstrate that BR signaling converges with auxin-mediated PdC3H17 to regulate xylem formation in *Populus* and thus provide insight into the regulation mechanism of BRs and the crosstalk with auxin signaling on xylem formation.

## Introduction

Xylem is a highly specialized vascular tissue with the roles of transporting water and minerals and also providing mechanical support for upright growth. The formation of xylem is mainly dependent on the activity of (pro)cambium and the rate of differentiation from it (Jouannet et al., 2015; Kondo et al., 2015). Several hormonal signals have been shown to be involved in maintenance of cambium and the formation of secondary vascular tissues, such as auxin, cytokinin, and gibberellin (Ragni et al., 2011; Ruzicka et al., 2015; Schaller et al., 2015). These hormones have been suggested to have different internal concentration gradients across wood-forming tissues, and these gradients are highly related with the gene expression and different developmental stages during the formation of secondary vascular tissues (Bhalerao and Fischer, 2014; Immanen et al., 2016).

Brassinosteroids (BRs) are also demonstrated to be involved in vascular differentiation (Yamamoto et al., 1997; Kang et al., 2017). Several studies in herbaceous plants have shown that BRs were implicated in the differentiation of primary vascular cell types. During the *in vitro* differentiation of tracheary elements (TEs) in the *Zinnia* cell cultures system, exogenous application of brassinolide (BL) could promote the formation of TEs by inducing the expression of genes related with secondary cell wall (SCW) deposition and programmed cell death (PCD; Yamamoto et al., 1997). Consistent with the idea that BRs were required for the later stages of TE differentiation, five different types of BRs accumulated both within the cells and in the TE culture medium during TE differentiation in *Zinnia* (Yamamoto et al., 2001). Moreover, the levels of BR intermediates have been revealed to peak at the transition from undifferentiated cells to TE (Yamamoto et al., 2001). In *Arabidopsis*, two BR receptor members BRL1 and BRL3, which were dominantly expressed in vascular tissues, could promote xylem differentiation and repress phloem cell differentiation (Cano-Delgado et al., 2004). As a key component in BR signaling, BES1 was revealed to function as a downstream target of GSK3s during xylem differentiation in a TDIF-dependent manner (Kondo et al., 2014), which also indicated that BES1 was a shared common signaling component between TDIF signaling pathway and the BR signaling pathway in xylem development (Ryu et al., 2010; Guo et al., 2013; Kondo et al., 2014). Another similar result was obtained using VISUAL (Vascular cell Induction culture System Using *Arabidopsis* Leaves) system in *Arabidopsis*, indicating that BES1 and its homolog BZR1 redundantly promoted both phloem and xylem differentiation (Saito et al., 2018). Notably, BES1 had a stronger impact on vascular cell differentiation than BZR1, suggesting that BES1 had a key role in BR signaling during vascular differentiation (Saito et al., 2018). However, the mechanism of BES1 in vascular development, especially its downstream components, remains largely unknown. Recent studies also revealed that BR signaling was closely related to secondary xylem formation in trees. A reduced lignification and altered cell-wall carbohydrate biosynthesis occurred within secondary xylem of *Liriodendron tulipifera* trees through the exogenous application of BR (Jin et al., 2014). In poplar, BR could induce the expression of *BEE3*, which encoded a basic helix–loop–helix transcription factor BR enhanced expression 3, resulting in enhanced secondary xylem formation (Noh et al., 2015). In addition, overexpression of several BR biosynthesis genes, such as *CYP85A3*, *DWF4*, and *DET2*, all showed increased secondary xylem formation in poplar due to an elevated level of BRs *in vivo* (Jin et al., 2017; Shen et al., 2018; Du et al., 2020; Fan et al., 2020). Recently, a key BR biosynthesis gene, *PtiCYP85A3*, was reported to play a critical role in BR-mediated tension wood formation in poplar (Jin et al., 2020). These studies suggest that BR signaling plays an important role in secondary growth and wood formation.

In addition, BRs also interplay with other hormones such as auxin during vascular differentiation (Hardtke, 2007; Sorce et al., 2013). In primary growth, BRs act in concert with auxin during the patterning of vascular bundles in the stem (Reinhardt et al., 2003; Sauer et al., 2006). BRs were predicted to control the bundle number by promoting the early cell divisions of procambial. In contrast, auxin maxima had a crucial role in defining spacing of the vascular bundle (Ibanes et al., 2009). But so far, the key components especially transcriptional factors (TFs) involved in BR signaling and the linkage with other hormones during secondary xylem formation are far from revealed.

The CCCH proteins possess a CCCH zinc finger motif composed of three cysteines followed by one histidine and have been found widely in eukaryotes (Fu and Blackshear, 2017). This type of proteins is conventionally known to be involved in functioning at both the transcriptional and posttranscriptional levels in animals (Lai et al., 2000; Lai and Blackshear, 2001; Hudson et al., 2004). Functional studies have revealed that plant CCCH zinc finger proteins play key roles in plant growth, hormone responses, and biotic/abiotic stress responses (Kim et al., 2008; Seok et al., 2016; Zhang et al., 2019). Our recent work revealed that CCCH protein PdC3H17 could form a module with its partner PdMYB199 driven by auxin in xylem formation (Tang et al., 2020). However, the connections between CCCH TFs and BRs during xylem formation have not yet been studied. It is noteworthy that such a connection is likely to exist, as RNA-seq data indicated that some cell differentiation and SCW biosynthesis genes were not only influenced by BR signaling, but also regulated by PdC3H17 (Du et al., 2020; Tang et al., 2020).

In this study, we tested the hypothesis that PdC3H17 interacted with BR signaling pathway in the modulation of xylem formation. Our results indicated that the function of PdC3H17 on xylem cell differentiation was mediated by BR signaling. Biochemical and molecular analysis indicated that *PdC3H17* acted as a downstream TF of PdBES1. The exogenous propiconazole (PCZ) treatment revealed that PdC3H17 was a positive regulator in xylem formation since the *PdC3H17DR* plants (dominant repression) displayed lower sensitivity to BR. Further, inhibition of BR synthesis reduced auxin sensitivity differences on xylogenesis between *PdC3H17DR* and overexpression plants, suggesting that PdC3H17 might be a co-effector linking auxin and BR signaling during secondary xylem formation.

## Materials and Methods

### Plant Materials and Growth Conditions

*Populus* “*Nanlin 895*” was used in this study and as the background of *PdC3H17* dominant negative and overexpression transformation. The *35S:FLAG-PdC3H17* (*PdC3H17OE*) and *PdC3H17*-*DR* transgenic plants used in this study were described in our previous reports (Chai et al., 2014a; Tang et al., 2020) and were grown in sterilized tissue culture bottle at 22°C under long-day conditions (16-h light/8-h dark).

*Arabidopsis thaliana* ecotype *Columbia* (Col-0) was used in this study for analysis of protoplast transcription activation activity, which were grown in soil at 22°C under long-day conditions (16-h light/8-h dark).

### Hormone Treatment Analyses

Four-week-old wild-type (WT) *Populus* “*Nanlin 895*” were incubated with the hormone solutions. The concentrations for the treatments were 0 (control), 1, 10, and 50 μM BL (Epibrassinolide; Sigma–Aldrich, St. Louis, MO, United States). BL-treated stems were subjected to *PdC3H17* expression analysis by quantitative real-time polymerase chain reaction (qRT-PCR). Three biological replicates were performed independently.

To analyze the responsiveness of BRs or auxin with BRs on xylem formation, at least three independent lines of root-removed control and transgenic seedlings were grown on the 1/2 MS medium with or without PCZ/(PCZ + IBA) at various determined concentrations for 21 days (8 h for gene expression analysis), and 0.3-cm segments were taken from their basal stems. These segments were fixed in 4% paraformaldehyde (Sigma-Aldrich) at 4°C for 4 days, dehydrated in graded ethanol series, and embedded into paraplast. The 5-μm sections were obtained using a Leica RM2235 rotary microtome and adhered to Superfrost microscope slides (Thermo). The sections were stained with 0.1% toluidine blue and observed using an Olympus X51 light microscope (Olympus). Radial widths of xylem were measured in three independent replicates using the SmileView software (JEOL).

### Real-Time Quantitative PCR

Total RNA was extracted from hormone-treated poplar stems or various tissues of 4-month-old WT *Populus* “*Nanlin 895*,” and first-strand cDNA syntheses were carried out as described previously (Li et al., 2018). qRT-PCR assays were performed on a LightCycler^®^ 480 Detection System (Roche) with TransStart Green qPCR superMix (TransGen Biotech). *PdUBQ10* was used as an internal control, and data represent the average of three biological replicates. Primers of the related genes are listed in Supplementary Table S1.

### Transient Gene Expression Assays

*Arabidopsis* leaf protoplasts transient assays were carried out following the procedure described previously (Tang et al., 2015). A 500-bp *PdC3H17* promoter sequence (including E-box mutation) was ligated upstream of the β-glucuronidase (GUS) reporter after removing the 35S promoter in pBI221 vector to create the reporter constructs. The *PdBES1* coding region was ligated between the 35S promoter and the *NOS* terminator after removing GUS from the pBI221 vector to create the effector construct. The reporter and effector constructs as well as the *35S:LUC* vector (internal control) were co-transformed into *Arabidopsis* leaf protoplasts. Samples were incubated overnight in darkness and then conducted the GUS and luciferase enzymatic assays. The reporter gene expression levels were determined as the relative ratio of GUS to luciferase activity. Data were the means of three biological replications.

### *In situ* Analysis

The 163-, 125-, and 143-bp CDS regions of *PdC3H17*, *PdBES1* (Potri.014G041600), and *PdBZR1* (Potri:011G106800) were separately used to synthesize the digoxigenin-labeled antisense and sense RNA probes with DIG RNA Labeling mix (Roche). The specific primers are listed in Supplementary Table S1. The 10th internode of 1.5-m-high *Populus* “*Nanlin 895*” plants growing in a glasshouse was sectioned, fixed in 4% glutaraldehyde, and embedded in paraffin. Stem section (8 μm) was cut on a rotary Leica RM 6025 microtome (Leica), mounted onto Superfrost glass slides (Thermo Fisher), and hybridized with the antisense or sense RNA probes. Hybridization and immunological detection were carried out according to the previous description (Xu C. et al., 2019).

### Yeast One-Hybrid Assay

The yeast one-hybrid assay was conducted as described previously (Wang et al., 2020). The coding sequence of *PdBES1* was inserted into pGADT7 vector to form pGADT7-BES1. The three tandem repeats of the E-box (CANNTG) motif in *PdC3H17* promoter were combined to pHIS2.1 to form bait reporter vector. Then, pGADT7-BES1 and pHIS-E-box (pHIS-Ev as negative control) vectors were transferred to yeast–one-hybrid yeast strain, followed by culture on SD/-Leu/-Trp medium for 3 days. Positive clones were then spread on SD/-Leu/-Trp and SD/-Leu/-Trp/-His (15 mM 3AT) medium, respectively.

### EMSA Analysis

The *PdBES1* coding region was fused in frame with MBP in the pMAL-p4x vector. Recombinant protein was expressed and purified from *Escherichia coli* using amylose resin (NEB). Biotin-labeled synthetic oligonucleotide was annealed with unlabeled oligonucleotides and then used as probes. A DNA protein–binding reaction was performed using a LightShift^®^ Chemiluminescent EMSA Kit (Pierce) according to the previous description (Xu Z. et al., 2019). Briefly, the labeled DNA fragments were incubated for 25 min with 180 ng of the recombinant protein in binding buffer [10 mM Tris (pH 7.5), 1 mM DTT, 2.5% glycerol, 50 mM KCL, 10 mM MgCl_2_, 0.1% Nonidet P-40, and 50 ng/μL poly (dI-dC)]. The protein-DNA fragments were separated from the unbound fragments by polyacrylamide gel electrophoresis. The DNA was electroblotted onto a nitrocellulose membrane and detected by chemiluminescence. The experiments were performed for three independent times.

## Results

### *PdC3H17* Is a Direct Target of PdBES1

**FIGURE 1:**
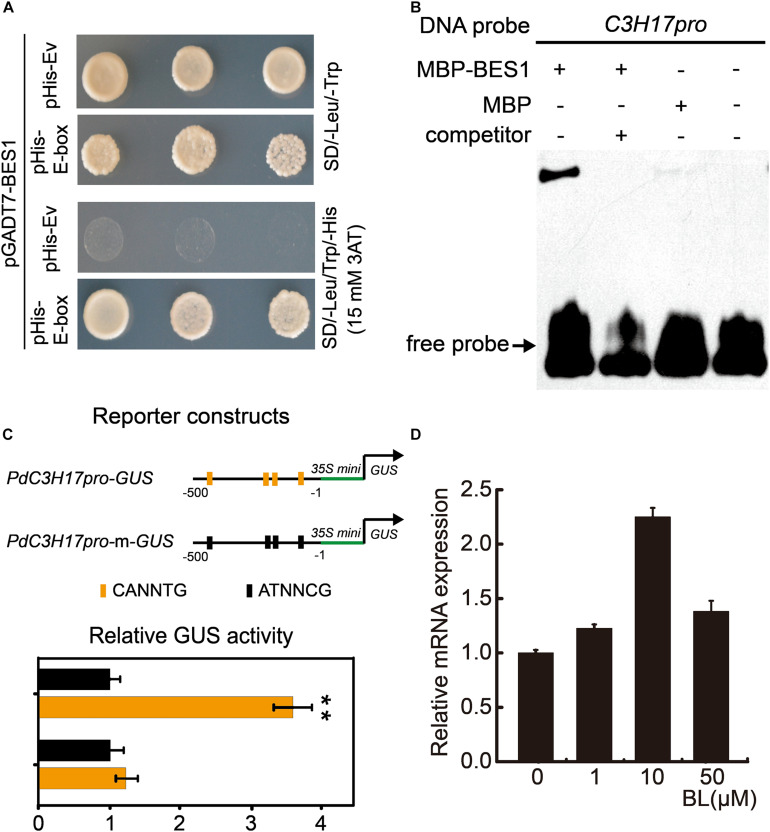
PdBES1 directly activates *PdC3H17 in vitro*. **(A)** Y1H analysis of PdBES1 binding to the *PdC3H17* promoter sequence indicated in Supplementary Figure S1. pHis-E-box showed recombinant pHis vector contained E-box (CANNTG) motif in *PdC3H17* promoter, pHis-Ev represented pHis empty vector. **(B)** EMSA analysis showed the specific binding of PdBES1 protein to the promoter fragment of *PdC3H17 in vitro*. **(C)** Transcription activity assay showed that PdBES1 activated the *PdC3H17* promoter-driven expression of the GUS reporter gene. For reporters, a short 500-bp promoter fragment of *PdC3H17* was constructed to drive the expression of GUS. The E-box harbored in these promoter fragments was disrupted via site-directed mutagenesis for generating *PdC3H17pro-m-GUS*. The effector encodes PdBES1 driven by the CaMV 35S promoter. The relative ratio of GUS to luciferase activity after cotransformation into *Arabidopsis* protoplast cells with different reporter and effector construct combinations was tested. Values for blank effector were normalized to 1. Error bars represent SD. Student *t-*test was performed to evaluate significant difference between values of blank effector and those of PdBES1 (*t*-test: **P < 0.01, *n* = 3). **(D)** Transcript levels of *PdC3H17* are shown after application of various concentrations of 24-eBL for 4 h. *PdUBQ10* was used as an internal control.

Previous study has demonstrated that *PdC3H17* was involved in xylem formation by interacting with auxin signaling (Tang et al., 2020). To identify the putative upstream components that regulate *PdC3H17*, a yeast one-hybrid (Y1H) screen assay was performed using poplar xylem–specific cDNA library. As bait, a 102-bp *PdC3H17* promoter segment including various potential binding sites analyzed in NCBI was fused to pHIS2.1 vector. A total of 18 xylem-expressed genes were found to possibly bind to the *PdC3H17* promoter sequence (Supplementary Table S2). Among these candidates, Potri.014G041600 represented the most promising one since its homolog in *Arabidopsis* was AtBES1, which plays a central role in the regulation of xylem formation in BR signaling. We thus named Potri.014G041600 as PdBES1. In order to determine whether *PdC3H17* could be a target of PdBES1, we first conducted *PdC3H17* promoter sequence analysis; the result showed that multiple E-box elements (CANNTG), which are putative BES1 binding site, were found within -500 bp relative to the transcription start site (Supplementary Figure S1). We then performed yeast one-hybrid assays and detection of strong reporter gene activation in yeast co-expressing AD-PdBES1 and PHis2.1-*C3H17pro* in Y1H system, which indicated that PdBES1 may have a connection with these binding sites located in *PdC3H17* promoter (Figure 1A). To further confirm this observation, we then purified PdBES1 protein fused with MBP tag from *E. coli* and conducted EMSA analysis *in vitro*. As shown in Figure 1B, PdBES1 specifically bound to *PdC3H17* promoter fragment (Figure 1B). To validate direct regulation of PdBES1 on *PdC3H17* via binding to these E-box elements, short promoter fragments harboring these E-box elements were constructed to drive the GUS reporter for transcriptional activation assays. Consistently, PdBES1 significantly activated the expression of GUS reporter driven by the E-box–harbored promoter fragments of *PdC3H17.* By contrast, the PdBES1-driven activation disappeared when the E-box elements harbored in the promoter fragments of *PdC3H17* were disrupted by site-directed mutagenesis (Figure 1C). The direct regulation of PdBES1 on *PdC3H17* prompted us to ask whether there is a connection between PdC3H17 and BR signaling. We then examined the expression profiles of *PdC3H17* in poplar plantlets (grown for 4 weeks *in vitro*) at various concentrations of BL (the most active BR). Total RNA was extracted from the BL-treated stems and subjected to the qRT-PCR using *PdC3H17*-specific primers. The results showed that the transcript level of *PdC3H17* was almost not changed after 1 μM BL treatment but was induced approximately twofold after 10 μM BL treatment. However, its expression was decreased again after 50 μM BL treatment (Figure 1D). These results indicated that PdBES1 could directly target *PdC3H17* and induce its expression in BR signaling pathway.

**FIGURE 2:**
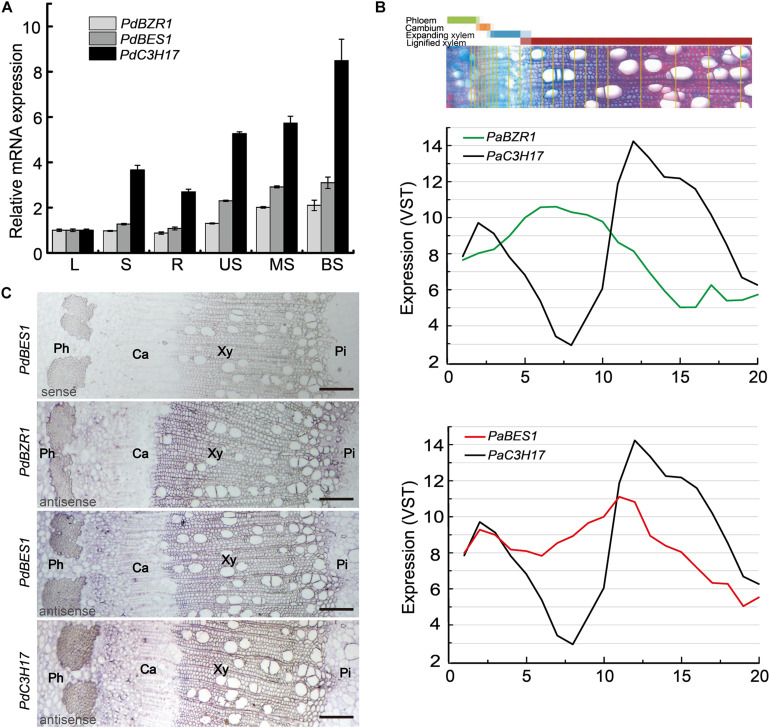
The comparison of expression patterns of *PdBZR1*, *PdBES1*, and *PdC3H17*. **(A)** The transcript levels of *PdBZR1*, *PdBES1*, and *PdC3H17* were measured in various poplar tissues. L: leaf; S: shoot; R: root; US: upper stem; MS: middle stem; BS: basal stem. Data are means ± SD of three biological repeats. **(B)** Expression patterns of *PdBZR1*, *PdBES1*, and *PdC3H17* genes across secondary stem tissues based on the AspWood RNAseq database (Sundell et al., 2017). The curves were generated using the values for the cryosection of stem tissues. The number in *x* axis showed continuous cryosection from phloem to lignified xylem. **(C)** *In situ* hybridization assay of *PdBZR1*, *PdBES1*, and *PdC3H17* in poplar stem. Cross-sections of the basal stems were hybridized with digoxigenin-labeled antisense RNA probes of *PdBZR1*, *PdBES1*, and *PdC3H17* or with a digoxigenin-labeled sense *PdBES1* RNA probe as a control. The hybridization signals are shown in purple. Ph: phloem; Ca: cambium; Xy: xylem; Pi: pith. Bars: 100 μm.

Since *PdC3H17* could be a target of PdBES1 *in vitro* and then whether there is a connection between these two genes *in vivo* during xylem formation. We explored it through detecting their expression profiles in wood-forming tissues because the function of *PdBES1* in xylem formation has not yet been studied. Except for *PdBES1*, as comparison, we also analyzed the expression patterns of *PdBZR1* (Potri.011G106800), homologous gene of *PdBES1* in BR signaling pathway. Tissue-specific expression analysis showed that *PdBZR1* and *PdBES1* were expressed in all tested tissues, which were consistent with their homologies in *Arabidopsis* (Yin et al., 2002; He et al., 2005). Besides, in accordance with the expression pattern of *PdC3H17*, *PdBZR1/BES1* were highly expressed in the middle and basal portion of stems, where the cells are undergoing secondary wall thickening and xylem formation (Figure 2A). Further, the continuous assays across secondary xylem tissues in poplar stems allowed us to evaluate spatial expression patterns of these highly wood tissue expressed genes (Sundell et al., 2017). By using this database, a highly similar expression pattern of *PdC3H17* and *PdBES1* was obtained, with two expression peaks in cambium zone and differentiated xylem zone. By contrast, low similarity across the wood-forming tissues was revealed between *PdC3H17* and *PdBZR1* (Figure 2B), which suggested a potential connection between *PdC3H17* and *PdBES1* during xylem formation.

To obtain detailed expression patterns of *PdBZR1/BES1* in secondary vascular tissues, RNA *in situ* hybridization was performed using the fourth internode of 1.5-month-old poplar (Figure 2C). The transcripts of *PdBES1* were preferentially accumulated in cambial zone and closely neighboring cell layers of the wood-developing stem, which was almost identical with the observation of distribution of *PdC3H17* across the stem with predominance expression in cambium adjacent cells toward xylem differentiation (Figure 2C). However, *PdBZR1* was distributed only in developing xylem, which was consistent with the analysis from Sundell and colleagues’ data. Taken together, the similar expression patterns indicated that *PdC3H17* could be involved in BR signaling pathway downstream of PdBES1 during xylem formation.

### *PdC3H17* Transgenic Plants Show Altered Response to BR During Xylem Formation

**FIGURE 3:**
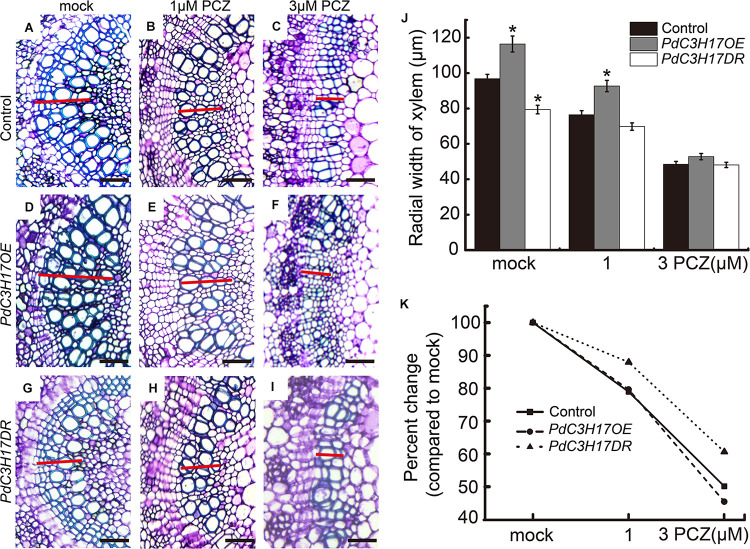
*PdC3H17* overexpression and dominant repressor plants show altered brassinosteroid (BR) response phenotypes during xylem formation. **(A–I)** BR responsiveness assays in the developing stems (xylem) of *PdC3H17* transgenic plants. Microscopic analysis of basal stem sections of the control **(A–C)**, *PdC3H17OE* **(D–F)**, and *PdC3H17DR* **(G–I)** plants grown in 1/2 MS medium containing 0, 1, and 3 μM propiconazole (PCZ), respectively, for 21 days. The red lines indicate the width of xylem, bars: 50 μm. **(J)** Statistical analysis of the radial width of xylem in the stems of the control and *PdC3H17* transgenic plants treated with PCZ in **(A–I)**. **(K)** Percent changes in xylem width compared with plants of the same genotype grown with no PCZ treatment. For each construct, at least three independent lines were used for measurement of xylem radial widths, and three biological replicates were performed independently. *t*-test: *P < 0.05.

**FIGURE 4:**
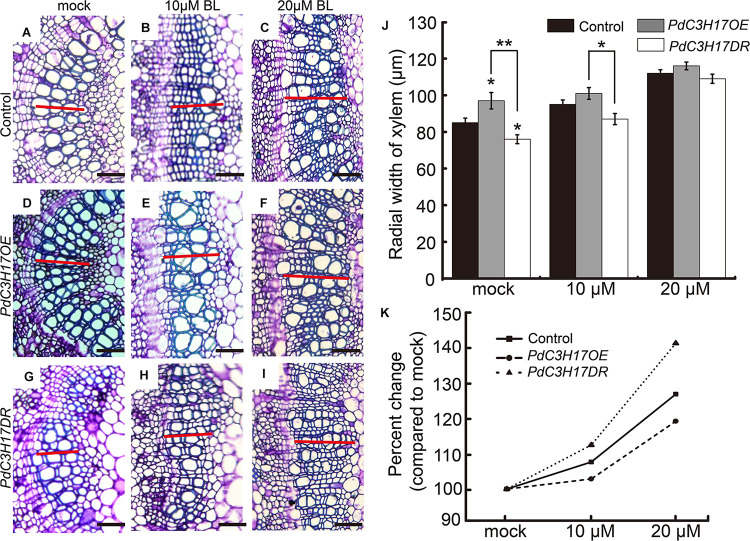
The xylem formation responses to brassinolide (BL) treatment in *PdC3H17* transgenic plants. **(A–I)** Brassinosteroid (BR) responsiveness assays in the developing stems (xylem) of *PdC3H17* transgenic plants. Microscopic analysis of basal stem sections of the control **(A–C)**, *PdC3H17OE* **(D–F)**, and *PdC3H17DR* **(G–I)** plants grown in medium containing 0, 10, and 20 μM BL for 21 days. The red lines indicate the width of xylem, bars: 50 μm. **(J)** Statistical analysis of the radial width of xylem in the stems of the control and *PdC3H17* transgenic plants treated with BL in **(A–I)**. For each construct, at least three independent lines were used for measurement of xylem radial widths, and three biological replicates were performed independently. *t*-test: *P < 0.05, **P < 0.01. **(K)** Percent changes in xylem width compared with plants of the same genotype grown with no BL treatment.

To evaluate the effect of BRs on the xylem formation in *PdC3H17* transgenic plants, a dose–response curve was generated for control (*Nanlin 895*), *PdC3H17OE*, and *PdC3H17DR* plants grown in the presence of various concentrations of exogenous PCZ (a BR synthesis inhibitor), which generally inhibits secondary xylem formation in stems (Du et al., 2020). The results showed that the xylem formation was repressed in all three genotypes when treated with PCZ, which was in agreement with the previous study (Du et al., 2020), but the transgenic plants exhibited different responses to PCZ as compared with the control. The *PdC3H17DR* plants had smaller reduced xylem formation compared to the control. More significantly, the *PdC3H17DR* plants produced almost the same xylem as in the control ones in the presence of 3 μM PCZ, whereas the xylem formation in overexpression plants decreased more drastically compared with the control ones (Figure 3). The results indicated that *PdC3H17DR* plants were less sensitive to inhibition of xylem formation caused by PCZ treatment, whereas *PdC3H17OE* plants were more sensitive (Figure 3K). This suggested that BRs were important for modulation of xylem formation by PdC3H17. To further test this hypothesis, we also generated dose–response curves for all three genotypes grown in the presence of BL. As expected, BL treatment had the opposite effect as compared with PCZ (Figure 4). Xylem formation was promoted by BL, and the promotion effect was more significant in *PdC3H17DR* as compared with the WT. However, the effect of BL was less significant in *PdC3H17OE* plants (Figure 4K). These results further suggested that there was a connection between PdC3H17 function and BR signaling during xylem formation.

### BR Signaling Regulates a Subset of PdC3H17-Mediated Xylem-Related Genes

**FIGURE 5:**
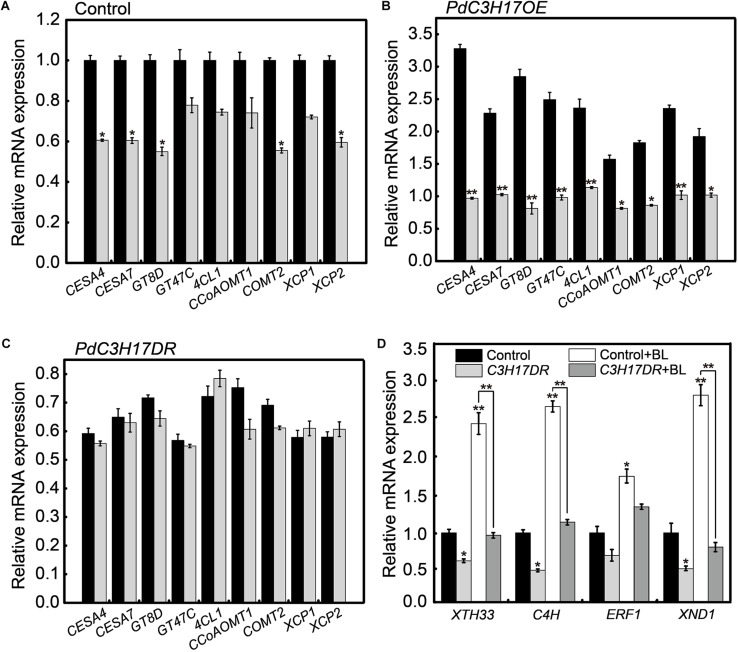
Brassinosteroid (BR) affects the expression of a subset of PdC3H17-mediated xylem-related genes. **(A–C)** qRT-PCR analysis of several PdC3H17-mediated xylem-related gene expression in control **(A)**, *PdC3H17OE* **(B)**, and *PdC3H17DR* **(C)** plants treated with no (black column) and 3 μM propiconazole (PCZ; gray column) for 8 h. The expression values of these genes in control **(A)** with no PCZ treatment were set to 1. *t*-test: *P < 0.05, **P < 0.01. **(D)** BR-induced gene expression is reduced in *PdC3H17DR* plants with or without brassinolide (BL) treatment. Data are means ± SD of three biological replicates. *t*-test: *P < 0.05, **P < 0.01.

Our previous study revealed that PdC3H17 affected xylem formation through modulation of SCW deposition and PCD (Chai et al., 2014a; Tang et al., 2020). One possible explanation for the results observed in the xylem formation assays may be that BR signaling affected PdC3H17 regulation of downstream xylem-related genes. To test this, gene expression was examined in seedlings grown in the presence or absence of PCZ using qRT-PCR. The expression of these xylem-related genes was decreased in both control and *PdC3H17OE* plants grown in the presence of PCZ compared with the MeoH mock, although this decrease was not statistically significant in control plants (Figures 5A,B). In contrast, no decrease of this gene expression was observed in *PdC3H17DR* plants grown in the presence of PCZ (Figure 5C). A battery of poplar MYB transcription factors that are involved in the transcriptional network mediated by MYB3/21 has previously been shown to play a key role in secondary xylem formation (Zhong et al., 2011), and these TFs were demonstrated to be regulated in *PdC3H17OE* and *PdC3H17DR* plants (Chai et al., 2014a). Therefore, we investigated whether these TFs transcripts are affected by inhibition of BR synthesis. The qRT-PCR results showed that of all six TFs, only the expression of *PdMYB121*, *PdMYB125*, and *PdMYB167* changed significantly after PCZ treatment, the PCZ treatment reduced the differences of these gene expression level among all three genotypes. However, expression levels of other genes had no obvious change in transgenic or control plants after PCZ treatment, suggesting that a unique transcriptional mechanism mediated by BR signaling might exist to impact xylem formation in *PdC3H17* transgenic lines (Supplementary Figure S2). These results indicated that BR signaling functioned to modulate the expression of xylem-related genes in *PdC3H17* transgenic plants.

We also examined several genes expression regulated by both BR and PdC3H17 in *PdC3H17DR* plants, including *XTH33*, *C4H*, *XND1*, and *ERF1* (Du et al., 2020; Tang et al., 2020). In general, the expression levels of these genes were decreased either in the absence or presence of BL in the *PdC3H17DR* plants compared to the control ones (Figure 5D). The BL induction was significantly decreased in at least three test genes (*XTH33*, *C4H*, and *XND1*). Taken together, the gene expression studies suggested that BR signaling was essential for the optimal expression of a subset of PdC3H17 downstream components during xylem formation.

### BR Converges With Auxin Signaling to Impact Xylem Formation in *PdC3H17* Transgenic Plants

**FIGURE 6:**
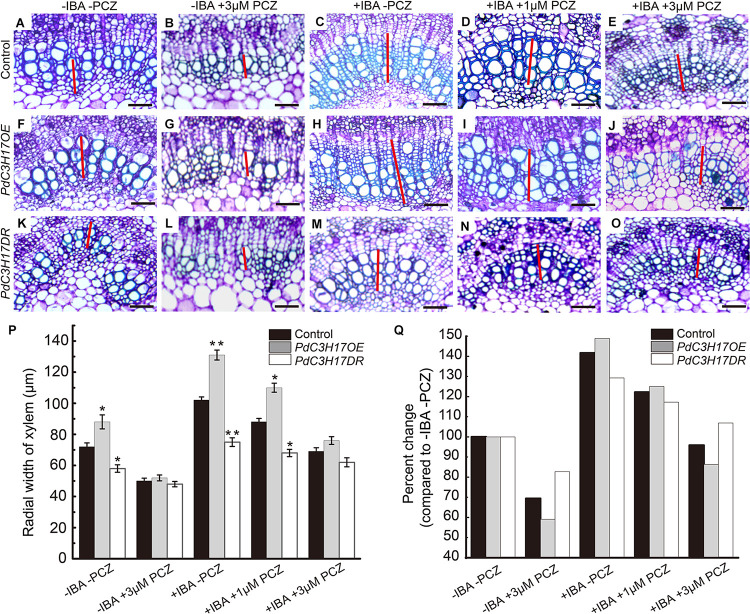
Propiconazole (PCZ) reduces the difference of auxin sensitivity in xylem formation of *PdC3H17* transgenic plants. **(A–O)** The effect of inhibition of brassinosteroid (BR) signaling on auxin responsiveness assays in the developing stems (xylem) of *PdC3H17* transgenic plants. Microscopic analysis of basal stem sections of the control **(A–E)**, *PdC3H17OE* **(F–J)**, and *PdC3H17DR* **(K–O)** plants grown in 1/2 MS medium containing 0 (mock), 3 μM PCZ, 2 mg/L IBA, 2 mg/L IBA + 1 μM PCZ, and 2 mg/L IBA + 3 μM PCZ for 21 days, respectively. The red lines indicate the width of xylem, bars: 50 μm. **(P)** Statistical analysis of the radial width of xylem in the stems of the control and *PdC3H17* transgenic plants in **(A–O)**. For each construct, at least three independent lines were used for measurement of xylem radial widths, and three biological replicates were performed independently. *t*-test: *P < 0.05, **P < 0.01. **(Q)** Percent change in xylem width compared with plant of the same genotype growing with no treatment.

*PdC3H17* was demonstrated to promote xylem formation at least in part by the control of auxin signaling pathway in our previous study (Tang et al., 2020). Multiple auxin and BR signaling components physically interact with each other and synergistically affect various physiological processes, such as cell elongation and division, vascular differentiation, and root development (Bao et al., 2004; Woodward and Bartel, 2005). Therefore, we hypothesized that the altered sensitivities of *PdC3H17DR* and *PdC3H17OE* plants to auxin during xylem formation might be related to C3H17’s impact on BR signaling. In order to test this hypothesis, we investigated xylem formation phenotypes of *PdC3H17* transgenic seedlings grown in 1/2 MS medium containing 2 μM IBA (the active form of auxin) and various concentrations of PCZ. The results showed that IBA treatment could significantly promote xylem formation in *PdC3H17OE* plant compared with control and *PdC3H17DR* plants, which was consistent with the previous study (Tang et al., 2020). Interestingly, we found that PCZ effectively blocked the enhanced sensitivity of *PdC3H17OE* plants to auxin (Figures 6F–J). Most notably, there was almost no difference in xylem formation between control and *PdC3H17OE* plants grown in the presence of 2 μM IBA and 3 μM PCZ (Figures 6E,J). In addition, PCZ treatment attenuated the defect xylem phenotype of *PdC3H17DR* plants observed in the presence of IBA. These results suggested that BR signaling was important for the auxin-associated xylem formation observed in the *PdC3H17* transgenic plants.

**FIGURE 7:**
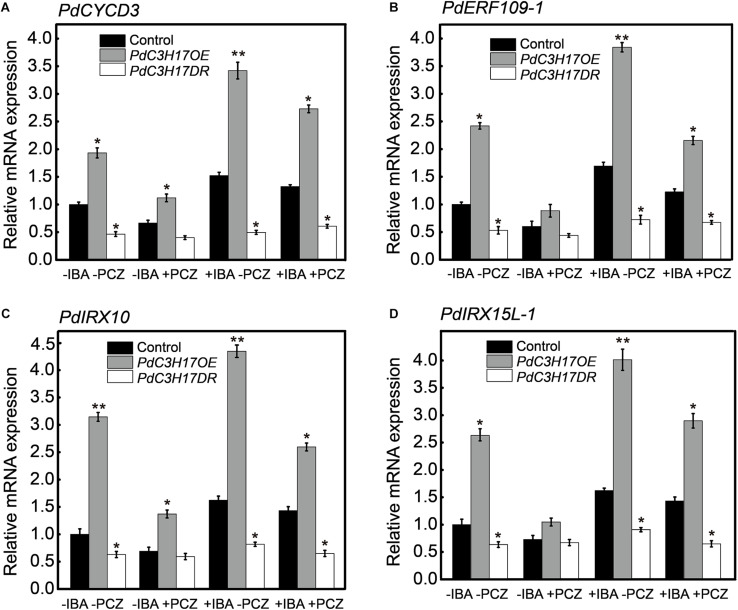
Brassinosteroid (BR) influences the transcription of auxin promotion of genes in *PdC3H17* transgenic plants. **(A–D)** qRT-PCR analysis of two auxin-mediated cell division related genes of *PdCYCD3* **(A)** and *PdERF109-1* **(B)**, two secondary cell wall deposition-related genes of *PdIRX10* **(C)**, and *PdIRX15L-1* **(D)** in *PdC3H17* transgenic plants treated with 0, 2 mg/L IBA, and 2 mg/L IBA + 3 μM propiconazole (PCZ) for 8 h, respectively. Data are means ± SD of three biological repeats. *t*-test: *P < 0.05, **P < 0.01.

As one of the outputs of auxin and BR signaling interaction, some transcription factors activated by these two different signaling pathways bind to the promoters of many shared target genes, synergistically regulating their transcription (Oh et al., 2014). In our previous study, we demonstrated that auxin-mediated PdC3H17-MYB199 module could promote xylem formation through the regulation of expression of genes related with xylem formation (Tang et al., 2020). In the current study, the inhibition of BR could attenuate the increased xylem formation driven by auxin in the *PdC3H17* transgenic plants (Figure 6). To investigate whether BR affects the expression of C3H17’s target genes in this process, the transcripts of four genes in our previous study (two genes of *CYCD3* and *ERF109-1* associated with cell division, two genes of *IRX10* and *IRX15L-1* associated with SCW deposition) were detected. The qRT-PCR analysis revealed that auxin enhanced PdC3H17-driven activation of the four genes’ expression (Figure 7), which was in accordance with the previous study, whereas PCZ treatment attenuated the expression of these genes driven by auxin (Figure 7). These results suggested that BR interacted with auxin signaling in promoting xylem formation through a co-regulation of downstream xylem-related genes mediated by PdC3H17.

## Discussion

Brassinosteroid has been recognized to be involved in xylem formation; many studies in *Arabidopsis* indicated that multiple components in BR signaling, including receptors and key TFs, have been revealed to differently regulate xylem formation (Cano-Delgado et al., 2004; Saito et al., 2018). Although we are starting to get a better insight into BR regulation of xylem formation in *Arabidopsis*, our understanding of the downstream regulation network of BR signaling during xylem formation in trees is limited. In this study, we characterized a CCCH protein PdC3H17, which has been demonstrated to have a role in xylem formation (Chai et al., 2014a), to participate in this process through involving in BR signaling pathway in poplar. Indeed, *PdC3H17* was markedly upregulated after BL treatment in poplar stem (Figure 1D), indicating that PdC3H17 had a possible link with BR in xylem formation. The following hormone treatment examinations further verified that BR has an important impact on PdC3H17-mediated xylem formation. Although our gene expression analyses suggested that the physiological results of this study were at least partially explained by the convergence of PdC3H17 and BR at the level of transcriptional regulation of genes encoding enzymes, which are directly involved in xylem formation, e.g., *CESAs* and *X* (Figure 5), there are other potential explanations for the altered sensitivities of the xylem formation in *PdC3H17* transgenic plants to BL and PCZ. For example, PdC3H17 could differentially regulate MYB gene pairs’ expression when the BR signaling was blocked (Supplementary Figure S2). Populus MYB gene pairs, such as *PdMYB90/167* and *PdMYB92/125*, were identified to coordinately regulate secondary wall deposition, as well as affected by PdC3H17 during xylem formation (Chai et al., 2014a,b). The different expression patterns in Supplementary Figure S2 indicated that BR might be associated with different pathways to modulate these C3H17-mediated gene pairs. Additionally, the xylem formation in *PdC3H17OE* showed less sensitivity to BR in our study (Figure 4), probably because the endogenous transcript of *PdC3H17* induced by BR treatment in *PdC3H17OE* was not as much as in the control and *PdC3H17DR* due to feedback inhibition. On the contrary, the PCZ treatment had the opposite effect as compared with BL in *PdC3H17OE*, and the xylem formation in *PdC3H17OE* was drastically suppressed compared with control (Figure 3). One possible explanation for the results may be that BR has a posttranscriptional regulation on PdC3H17 besides the transcriptional regulation, and the functional PdC3H17 may require appropriate BR level. This type of regulation was previously reported. For instance, the C3H-type transcription factor LIC antagonizes BZR1 to repress BR signaling in rice. *LIC* transcription was rapidly induced by BR treatment and was found to function as a dephosphorylation form at high BR level (Zhang et al., 2012).

As a key regulator in both BR and TIDIIF-GSKs signaling pathways, BES1 has an important role in promoting xylem formation in *Arabidopsis* (Saito et al., 2018), but its downstream regulation network is still unclear. *PdC3H17* was confirmed to be a target of PdMYB3/21, secondary top regulator in poplar xylem formation (Chai et al., 2014a). In the current study, we showed that *PdC3H17* could also be a target of PdBES1 in the BR signaling during xylem formation. The *in situ* assay revealed that *PdC3H17* and *PdBES1* had a highly overlapped expression profile within the wood-forming tissues, indicating a direct connection between these two genes. The subsequent molecular analyses including Y1H, transcription activation assays, and EMSA analysis further confirmed that *PdC3H17* was involved in BR signaling through the direct regulation by PdBES1 (Figure 1). Although the precise role of poplar PdBES1 in xylem formation has not been explored, the evidences in our study indicated that PdBES1 probably functioned in poplar xylem formation. For instance, PdBES1 shared more than 70% amino acid similarity with AtBES1, implying these two homologous proteins might be functionally conserved in xylem formation. Additionally, *PdBES1* had a higher transcript level in the stem in tissue expression analysis and was predominantly distributed in the cambium and developing xylem section according to *in situ* and AspWood RNAseq assays (Figure 2), revealing that PdBES1 has a potential role in xylem formation.

The crosstalk of BRs and auxin plays crucial roles in many aspects of plant growth and development, such as cell elongation, organ pattern formation, and vascular development (Woodward and Bartel, 2005; Altmann et al., 2020). For instance, SMOS1 and SMOS2/DLT integrate auxin and BR signalings to regulate lamina joint bending in rice (Hirano et al., 2017). Favero et al. (2017) revealed that *SOB3* could converge with BR and auxin signalings to influence hypocotyl growth. In addition, vascular-related unknown protein 1 (VUP1) was considered to regulate secondary wall formation during vascular development through modulating the expression of many BR- and auxin-responsive genes in *Arabidopsis* (Grienenberger and Douglas, 2014). In the current study, we showed a novel type of crosstalk between auxin and BR signaling occurred through PdC3H17 during xylem formation. Regulation of PdC3H17-mediated xylem formation by auxin signaling was previously reported by Tang et al. (2020) and further confirmed in this study. Moreover, the inhibition of BR signaling could obviously reduce the sensitivity of *PdC3H17* transgenic plants in auxin-mediated xylem formation (Figure 6). During the co-regulated biological processes, BR and auxin signaling shares many transcriptional target genes. For example, the SMOS1–SMOS2 complex regulates joint bending through the regulation of *OsPHI-1*, and *SOB3* modulates hypocotyl elongation mediated by auxin and BR through influencing *SAUR19* subfamily transcription (Favero et al., 2017; Hirano et al., 2017). In our results, the expression of some xylem-related genes affected by auxin in previous study was also changed by PCZ treatment, indicating these genes were common targets for the two hormones and were responsible for the alternation of xylem formation observed in *PdC3H17* transgenic plants (Figure 7).

Previous reports suggested that interactions between TFs involved in auxin and BR signaling might act as the points of auxin-BR crosstalk; one case is the response of *SAUR15* to BR and auxin. The expression of *SAUR15* depends on the combined binding of BES1 and MONOPTEROS/ARF5 within its promoter harboring a hormone up at dawn–type E-box and AuxRE *cis* elements, respectively (Walcher and Nemhauser, 2012). In our study, we also found that there were auxin response elements within promoter of *PdC3H17* (data not shown), which inferred that PdC3H17 could combine these two hormones through BES1 and other ARFs during xylem formation. Future studies should investigate which ARFs are involved in this interaction.

## Conclusion

Our study revealed *PdC3H17* was involved in BR signaling during xylem formation and might be a target of PdBES1 at the transcriptional level. Moreover, *PdC3H17* could also be a crosstalk point of the auxin and BR signaling pathways in this process. However, how these hormones act together to regulate vascular development need far more explored. Our findings provide new insights into the mechanism of BRs on xylem formation and the interaction with other hormones within this process.

## Data Availability Statement

Publicly available datasets were analyzed in this study. This data can be found here: AspWood, http://aspwood.popgenie.org.

## Author Contributions

XT, SL, and GZ designed research. XT, CW, YL, NM, and GH conducted the experiments and analyzed the data. XT and HX wrote the manuscript. SL, GC, HX, and GZ revised the manuscript. All authors read and approved the manuscript.

## Conflict of Interest

The authors declare that the research was conducted in the absence of any commercial or financial relationships that could be construed as a potential conflict of interest.

## Supplementary Material

Supplementary Figure 1 Diagram summarizing of cis-elements in the promoter region upstream of start code of *PdC3H17*.

Supplementary Figure 2 Brassinosteroid (BR) affects the expression of a subset of *PdC3H17*-mediated MYB transcription factors.

Supplementary Table 1 Primers used in this study.

Supplementary Table 2 List of putative candidate transcription factors regulating *PdC3H17* expression identified by yeast one-hybrid assay.
