# Supplementary material for: Brassinosteroid Signaling Converges With Auxin-Mediated C3H17 to Regulate Xylem Formation in Populus
Source: Front Plant Sci. 2020 Oct 27;11:586014. doi: 10.3389/fpls.2020.586014 (PMC7652770; doi:10.3389/fpls.2020.586014)
Supplement: Supplementary Figure 1 — Diagram summarizing of cis-elements in the promoter region upstream of start code of PdC3H17. [file Data_Sheet_1.PDF]

Title: Brassinosteroid signaling converges with auxin -mediated C3H17 to regulate the xylem formation in *Populus*

Xianfeng Tang[1], Congpeng Wang[2], Yu Liu[2], Guo He[1], Nana Ma[3], Guohua Chai[2], Shengjun Li[1], Hua Xu[1*] and Gongke Zhou[2, 1*]

Supplementary materials

Figure S1 BR affects the expression of a subset of PdC3H17-mediated MYB transcription factors.

Figure S2 Diagram summarizing of *cis*-elements in the promoter region upstream of start code of *PdC3H17*.

Table S1 Primers used in this study.

Table S2 List of putative candidate transcription factors regulating *PdC3H17* expression identified by yeast one-hybrid assay.

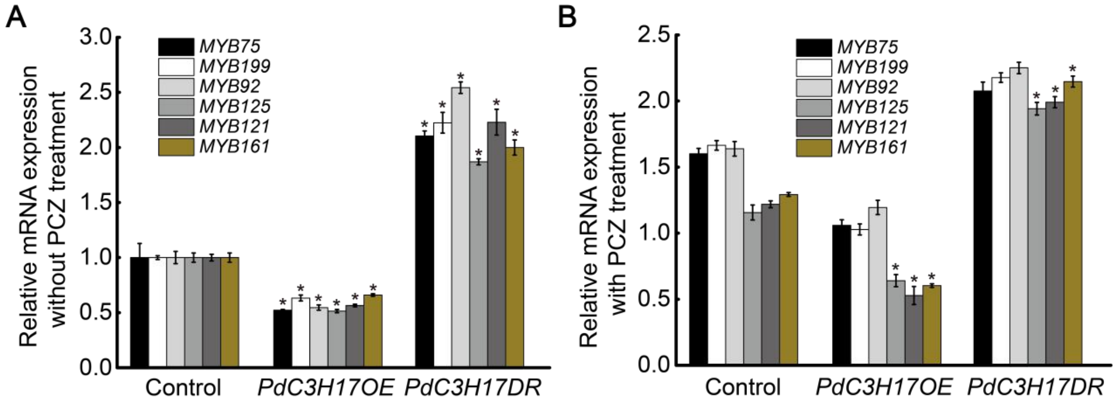

Figure S1. BR affects the expression of a subset of PdC3H17-mediated MYB transcription factors. qRT-PCR analysis of these MYB gene expression in control and *PdC3H17* transgenic plants without (**A**) and with (**B**) PCZ treatment for 8h. The expression values of these genes in control (**A**) with no PCZ treatment were set to 1. Data were means ± SD of three biological replicates. *t*-test: *P<0.05.

CTTCCTTCTTTGCCACTCCACCACCACCACCACCCACATCTC
CTCCTCCTCCTAACCACCTTTCCTTTTCTATCTAACTAATTAT
AATATATATACTCTCTCCCATTTGCATCCATTGCCTATCTCTC
TCTCTCTCTCTCCACTTTTGTTTTTTAATTTCTCTCTCTAAA
ACTGCTTTCTGTTTCTGTTACTGTTTCTCTAACGGTTTTCCAC
TTCCCCTGGCGGGAAACATACATCTTTTTGAAAGATTTTGCT
GTCGTGTTACTGCACCGTTTTCTCTCGCTACTCCCAACCAAT
AACTTGCATCCACGTGTTGAAAAAAGACAGATGAGTTCACCA
GGACGAACACGATTGGTTCTCACAAATTACCCATCATGATTT
GGTAAGTACCAGATGTAAGAGCCGGCGTTTATATATATATAT
ATATATATATATGCTGTAAAGAAGCTTCATCTCTCCCTCTCTC
TCTCTCTCTCTCTCTCTCTCTTCTATCAAGAAATG

Figure S2. Diagram summarizing of *cis*-elements in the promoter region upstream of start code of *PdC3H17*. ATG: the start coding site; CANNTG: the predicted E-box elements binding site by PdBES1; EMSA probe sequence; The underline indicated sequence linked into pHIS2.1 in Y1H analysis.

Table S1 Primers used in this study.

Gene Name	Accession number	Primers	Sequence(5'-3')	Goal
PdUBQ10	Potri.014G115100	PdUBQ10-F	AGACCTACACCAAGCCCAAGAAGAT	For detecting *PdUBQ* expression by qRT-PCR
		PdUBQ10-R	CCAGCACCGCACTCAGCATTAG	
PdC3H17	Potri.004G095100	PdC3H17qPCR-F	GCTCCTCTTAACCCAAAGGC	For amplifying transcript of *PdC3H17* by qRT-PCR
		PdC3H17qPCR-R	CACACCATCACCAGAATCCAGC	
PdCESA4	Potri.002G257900	PdCESA4qPCR-F	CACAGGTTATCCCACTTTTGCT	For amplifying transcript of *PdCESA4* by qRT-PCR
		PdCESA4qPCR-R	CATACGCTTGCTTGCTAACAGA	
PdCESA7	Potri.018G103900	PdCESA7qPCR-F	CAAGCAATGTGGACTCAACTGTTA	For amplifying transcript of *PdCESA7* by qRT-PCR
		PdCESA7qPCR-R	AAGCAGGATGCACATGTATCTTCT	
PdGT8D	Potri.001G416800	PdGT8DqPCR-F	CCTGTGGGCTAAGTACATCAACT	For amplifying transcript of *PdGT8D* by qRT-PCR
		PdGT8DqPCR-R	GCAGTCAACATTTTCATCTCAAA	
PdGT47C	Potri.009G006500	PdGT47CqPCR-F	AGGGAGAGGTTTCTGTTTTGAGA	For amplifying transcript of *PdGT47C* by qRT-PCR
		PdGT47CqPCR-R	AAATGGGTTGGGTTTTCAGTAAA	
Pd4CL1	Potri.001G036900	Pd4CL1qPCR-F	CCAGGCATATAACTGAAGACGTTA	For amplifying transcript of *Pd4CL1* by qRT-PCR
		Pd4CL1qPCR-R	GTTCTTACGTTTGGTACGTGTCTT	
PdCCoAOMT1	Potri.009G099800	PdCCoAOMT1qPCR-F	ACCTGCCAGTATTGTTATCTGATGT	For amplifying transcript of *PdCCoAOMT1* by qRT-PCR
		PdCCoAOMT1qPCR-R	CCATTGAAATACAAAGTGGGTAAAA	
PdCOMT2	Potri.012G006400	PdCOMT2qPCR-F	TGCTGCTGTCTCTGCTTTTGAT	For amplifying transcript of *PdCOMT2* by qRT-PCR
		PdCOMT2qPCR-R	GGAAGGCGGTGAAGTTATTTGA	
PdXCP1	Potri.004G207600	PdXCP1qPCR-F	TCTCAATCTCCATGGCACTCTCTGT	For amplifying transcript of *PdXCP1* by qRT-PCR
		PdXCP1qPCR-R	AGTCGTGGGCTAGAACCGAACACA	
PdXCP2	Potri.005G256000	PdXCP2qPCR-F	GCTCAAATGGCTCTCCCCGCA	For amplifying transcript of *PdXCP2* by qRT-PCR
		PdXCP2qPCR-R	AGACGTCAAGTCTTCTGGGGCA	

PdXND1	Potri.003G022800	PdXND1qPCR-F	GGGTCTATGAGCGGAGTTGTGAG	For amplifying transcript of *PdXND1* by qRT-PCR
		PdXND1qPCR-R	CTAGCCTCCACAAATAAGTTGAGGAC	
PdXTH33	Potri.014G115000	PdXTH33qPCR-F	TGCTAATCTTGCTCTTGACAAATC	For amplifying transcript of *PdXTH33* by qRT-PCR
		PdXTH33qPCR-R	TGCGGGTAATTATCTGCATTAGACA	
PdERF1	Potri.002G039100	PdERF1qPCR-F	TGGTACTGTTTAATGATTTAGGCGC	For amplifying transcript of *PdERF1* by qRT-PCR
		PdERF1qPCR-R	CCATGGCCGCCTCCTAACCCCTCTGT	
PdC4H	Potri.019G130700	PdC4HqPCR-F	AAGGAGGTTAAAGAGAGAAGGCTGCA	For amplifying transcript of *PdC4H* by qRT-PCR
		PdC4HqPCR-R	TCAAGCCTTCATTGCTCATGCTCTT	
PdBZR1	Potri.011G106800	PdBZR1qPCR-F	ACCACTTACCGCAAGGGATGCAAAC	For amplifying transcript of *PdBZR1* by qRT-PCR
		PdBZR1qPCR-R	GCATTAGCAGCATAGGAGGATGAAAC	
PdBES1	Potri:014G041600	PdBES1qPCR-F	TGAAGAAGATGGAACTACTTATCGC	For amplifying transcript of *PdBES1* by qRT-PCR
		PdBES1qPCR-R	TGGCTTGATAGGAAGGAATTGGACTA	
PdBZR1	Potri.011G106800	PdBZR1in situ-F	TGATCCAGGATGGTTTGCTGGGA	For amplifying CDS region of *PdBZR1* cDNA using for in situ hybridization
		PdBZR1in situ-R	AAGCCTCTTCCTTGAAGCCAAACG	
PdBES1	Potri:014G041600	PdBES1in situ-F	GGTTGGGTTGTTGAAGAAGATGGAA	For amplifying CDS region of *PdBES1* cDNA using for in situ hybridization
		PdBES1in situ-R	GGAAGGAATTGGACTAGGAAACAAC	
PdC3H17	Potri.004G095100	PdC3H17in situ-F	ATGGAGAAAACAGAATCACC	For amplifying CDS region of *PdC3H17* cDNA using for in situ hybridization
		PdC3H17in situ-R	AGAGCGAGTTCGGGAGCCGT	
PdBES1	Potri:014G041600	PdBES1TAA-F	GCTCTAGAATGACGTCAGATGGGGC	For amplifying *PdBES1* full-length cDNA for transcription activation
		PdBES1TAA-R	CGAGCTCGCTAACTCTGAGCCTTGCCAC	
PdC3H17	Potri.004G095100	PdC3H17TAApro-F	CTTCCTTCTTTGCCACTCCA	For amplifying the *PdC3H17* promoter fragment for transcription activation
		PdC3H17TAApro-R	TTCTTGATAGAAGAGAGAGAG	
PdBES1	Potri:014G041600	PdBES1AD-F	ATGACGTCAGATGGGGCAACCTCG	For amplifying *PdBES1* full-length cDNA for pGADT7-PdBES1 construction
		PdBES1AD-R	ACTCTGAGCCTTGCCACTTCCAAGTG	

PdC3H17	Potri.004G095100	PdC3H17PHIS2.1pro-F<br>PdC3H17PHIS2.1pro-R	TTGCATCCACGTGTTGAAAAAAG<br>CTCTTACATCTGGTACTTA	For amplifying *PdC3H17* promoter fragment for YIH analysis
PdBES1	Potri:014G041600	PdBES1EMSA-F<br>PdBES1EMSA-R	GAATTCATGACGTCAGATGGGGCAACC<br>GTCGACACTCTGAGCCTTGCCACTTCCA	For amplifying *PdBES1* fusioned with MBP tag protein for EMSA analysis
PdC3H17	Potri.004G095100	PdC3H17EMSApro-F<br>PdC3H17EMSApro-R	CATCCACGTGTTGAAAAAAGACAGATG<br>ACTCATCTGTCTTTTTTCAACACGTGGA	For amplifying *PdC3H17* probe for EMSA analysis
PdCYCD3	Potri.007G048300	PdCYCD3qPCR-F<br>PdCYCD3qPCR-R	CACGGAGAGGATTTCAGGTGGA<br>CAGCAGCAAACATATGGGTGAGACATC	For detecting *PdCYCD3* expression by qRT-PCR
PdERF109-1	Potri.004G141200	PdERF109-1qPCR-F<br>PdERF109-1qPCR-R	GGAGTTCAATAGTCTTAATGTGCCG<br>CCGACGATGGAGGCCATATTGAGAT	For detecting *PdERF109-1* expression by qRT-PCR
PdIRX10	Potri.001G068100	PdIRX10qPCR-F<br>PdIRX10qPCR-R	CACGGAGAGGATTTCAGGTGGA<br>CAGCAGCAAACATATGGGTGAGACATC	For detecting *PdIRX10* expression by qRT-PCR
PdIRX15L-1	Potri.005G141300	PdIRX15L-1qPCR-F<br>PdIRX15L-1qPCR-R	AACACAAAGCTGATCCTTCTTCATCC<br>GTCTCTCTCGTGTAGATTAGTGTAAGA	For detecting *PdIRX15L-1* expression by qRT-PCR
PdMYB75	Potri.015G129100	PdMYB75qPCR-F<br>PdMYB75qPCR-R	CATGCCTCCTCTCGTACTGAT<br>GACCATATGAAACTCATTGTTGGA	For amplifying transcript of *PdMYB75* by qRT-PCR
PdMYB92	Potri.001G118800	PdMYB92qPCR-F<br>PdMYB92qPCR-R	TAATTCCTCCGGCTGCGGCG<br>GCCCGAAGTCGTGAACGCCAA	For amplifying transcript of *PdMYB92* by qRT-PCR
PdMYB199	Potri.012G127700	PdMYB199qPCR-F<br>PdMYB199qPCR-R	GGGTGAGTGGGGCTTTGTTCTCTG<br>AGGGCCCTTCTTCACACCGAGTTT	For amplifying transcript of *PdMYB199* by qRT-PCR
PdMYB121	Potri.002G185900	PdMYB121qPCR-F<br>PdMYB121qPCR-R	TCCACGAGTAATTGCCCGCCC<br>GTTCGTCGCGAGCCTGGCAT	For amplifying transcript of *PdMYB121* by qRT-PCR
PdMYB125	Potri.003G114100	PdMYB125qPCR-F<br>PdMYB125qPCR-R	ACAAAATTATTCCTCTGGCCGCAGA<br>CAATCGAATCCGAAATCGTGAACGC	For amplifying transcript of *PdMYB125* by qRT-PCR
PdMYB161	Potri.007G134500	PdMYB161qPCR-F<br>PdMYB161qPCR-R	CTCCAAGTAAAGACCGAGTCTAG<br>ACCAGCCCTGCTCTTAGGC	For amplifying transcript of *PdMYB161* by qRT-PCR

Table S2 List of putative candidate transcription factors regulating *PdC3H17* expression identified by yeast one-hybrid assay.

Gene symbol/locus	Homolog in Arabidopsis	Other Names in Arabidopsis
Potri.014G041600	AT1G19350	BRASSINAZOLE-RESISTANT 2, BES1
Potri.002G042000	AT4G16430	BHLH03
Potri.007G046200	AT4g36920	ATAP2
Potri.013G108600	AT1g72050	TRANSCRIPTION FACTOR IIIA
Potri.011G058400	AT4G28500	SND2
Potri.002G038800	AT2G31200	ACTIN DEPOLYMERIZING FACTOR 6
Potri.002G094200	AT1G78080	ATWIND1
Potri.005G158100	AT5G50010	TRANSCRIPTION FACTOR BHLH145
Potri.001G267300	AT3G08500	MYB85
Potri.009G134900	AT2G16770	ATBZIP23
Potri.001G375800	AT3G15030	TCP TRANSCRIPTION FACTOR 4
Potri.004G159300	AT4G34610	BEL1-LIKE HOMEODOMAIN 6
Potri.007G048900	AT4G37260	MYB DOMAIN PROTEIN 73
Potri.009G053900	AT3G08500	MYB85
Potri.002G119400	AT4G37540	LBD 39
Potri.009G089600	AT3G49940	LBD 38
Potri.009G009800	AT2g27990	BEL1-LIKE HOMEODOMAIN 8
Potri.006G237500	AT4G32880	HOMEOBOX GENE 8
